# PSMA-targeted radioligand therapy in advanced prostate cancer: a narrative review of ^177Lu-PSMA, emerging ^225Ac-PSMA strategies, and therapeutic sequencing

**DOI:** 10.3389/or.2026.1834484

**Published:** 2026-07-15

**Authors:** Natalia Gierulska, Nina Jankowska, Adrianna Kwiatkowska, Piotr Kuchno, Kinga Łysak, Katarzyna Szklener, Kamil Kośmider, Magdalena Skórzewska

**Affiliations:** 1 Department of Clinical Oncology and Chemotherapy, Student Scientific Society, Medical University of Lublin, Lublin, Poland; 2 Copernicus Hospital, Gdańsk, Poland; 3 Department of Clinical Oncology and Chemotherapy, Medical University of Lublin, Lublin, Poland; 4 Department of Pneumonology, Oncology and Allergology, Medical University of Lublin, Lublin, Poland

**Keywords:** actinium-225, bone metastases, cancer, lutetium-177, nuclear medicine, prostate cancer, radioisotopes, radionuclides

## Abstract

Prostate cancer remains a leading cause of cancer-related morbidity and mortality among men, and metastatic castration-resistant prostate cancer continues to present substantial therapeutic challenges despite advances in androgen receptor pathway inhibitors, taxane chemotherapy, PARP inhibitors, and immunotherapy for selected molecular subgroups. Prostate-specific membrane antigen (PSMA)-targeted radioligand therapy has emerged as a clinically important theranostic strategy that combines molecular imaging-based patient selection with targeted delivery of cytotoxic radiation to PSMA-expressing tumor sites. This narrative review summarizes the biological rationale, clinical evidence, safety profile, and evolving treatment-sequencing considerations for PSMA-targeted radioligand therapy in advanced prostate cancer. Particular emphasis is placed on ^177Lu-PSMA-617, which has demonstrated improved radiographic progression-free survival and overall survival in pivotal randomized trials, including VISION, TheraP, and PSMAfore. The review also examines investigational alpha-emitting PSMA-targeted therapies, particularly ^225Ac-PSMA compounds, which offer high-linear-energy-transfer cytotoxicity and may have activity in selected patients after progression on beta-emitting radioligand therapy, although definitive phase III evidence remains lacking. The established bone-targeted alpha-emitter ^223Ra is discussed separately as contextual therapy for selected patients with symptomatic bone-predominant mCRPC without visceral metastases. As clinical use of radioligand therapy expands, optimization of PSMA PET-based patient selection, management of salivary, hematologic, and renal toxicities, sequencing with other systemic therapies, and integration into multidisciplinary care pathways will be essential. Future research should prioritize randomized clinical trials, individualized dosimetry, predictive biomarkers, toxicity mitigation, and patient-centered implementation strategies.

## 1 Introduction

Prostate cancer is commonly observed in men aged 45 to 60, yet peak incidence rates occur in elderly populations over the age of 65. Furthermore, when compared to White men, African-American men demonstrate a disproportionately higher incidence rate and are more susceptible to aggressive variants of the cancer. It is the second most common cancer and the fifth most common cause of cancer deaths in men worldwide (1, 2). In recent years, incidence rates of prostate cancer worldwide have increased. It is estimated that the mortality rates will also increase. Due to the widespread implementation of routine prostate-specific antigen (PSA) screenings for men over the age of 50 in developed regions like North America and Europe, prostate cancer is predominantly diagnosed at early, localized stages (TNM Stage I or II). Furthermore, most patients have a relatively indolent clinical course. Only a small percentage of cases (approximately 5%–10%) are diagnosed in advanced stages or with metastases (2). The early diagnosis is made primarily on the basis of a PSA level in peripheral blood. PSA is a glycoprotein that is secreted by the epithelial cells of the prostate gland. Historically, a rigid prostate-specific antigen (PSA) threshold of >4 ng/mL was utilized to prompt further diagnostics. However, contemporary clinical guidelines advocate for a risk-adapted approach to PSA interpretation. Rather than relying on a universal diagnostic cutoff, modern screening evaluates PSA levels in the context of multiple patient-specific variables, including age, prostate volume, race, family history, and prior testing trajectories. While PSA screening is instrumental in reducing prostate cancer mortality, these nuanced, multivariable diagnostic algorithms are essential to mitigate the risks of over-detection and the subsequent overtreatment of indolent disease (1).

In accordance with contemporary risk-adapted diagnostic pathways, multiparametric magnetic resonance imaging (mpMRI) has become the central imaging modality for prostate cancer evaluation prior to biopsy. It offers high sensitivity for detecting and localizing clinically significant lesions, directly enabling targeted biopsy approaches. While transrectal ultrasound was historically used for primary evaluation, its inferior sensitivity and lesion-characterization capabilities compared to mpMRI limit its current diagnostic role. Consequently, MRI-targeted core-needle biopsies, increasingly performed via the transperineal route in alignment with current guidelines to minimize infectious complications, remain the reference standard for histopathological confirmation. Beyond initial diagnosis, the application of prostate-specific membrane antigen positron emission tomography combined with computed tomography (PSMA PET/CT) is highly dependent on the patient’s specific clinical state. Current guidelines position PSMA PET/CT as the superior modality for the initial staging of unfavorable intermediate- and high-risk disease, as well as for localizing recurrence. Furthermore, it is essential for the precise assessment of metastatic disease and serves as a mandatory prerequisite for confirming target expression prior to the initiation of PSMA-targeted radioligand therapy (1, 3).

Over 95% of prostate cancers are adenocarcinomas arising from the glandular epithelium of the prostate, most commonly acinar adenocarcinoma (3). Risk factors include primarily a positive family history, age, race, obesity, environmental factors, and the occurrence of BRCA1 and BRCA2 mutations (4, 5). Management of the localized disease should be tailored according to the risk group. Generally, the European Society of Medical Oncology (ESMO) differentiates 3 different risk groups: low-, intermediate- or high-risk (6).

The stratification criteria are presented in Table 1 (6).

**TABLE 1:** Risk stratification of prostate cancer based on ESMO Clinical Practice Guidelines (6).

Risk groups of prostate cancer	TNM classification	Gleason score	PSA level (ng/mL)
Low risk	T1-T2a	≤6	≤10
Intermediate risk	T2b	= 7	10–20
High risk	≥ T2c	≥8	>20

Legend: TNM - Tumor, Nodules, Metastases, PSA - Prostate-Specific Antigen.

In the case of low risk localized prostate cancer, therapeutic strategies include: 1) radical prostatectomy; 2) radical radiotherapy - external beam radiation therapy (EBRT) or brachytherapy; 3) active surveillance; 4) watchful waiting, which is an option for men who do not want to have other treatments or they are not suitable for treatments. In cases of intermediate risk, the patient can additionally receive short-course neoadjuvant androgen deprivation therapy (ADT) for 4–6 months prior to the radiotherapy (EBRT or brachytherapy). Cases of high risk or locally advanced should be qualified for radical prostatectomy with pelvic lymphadenectomy or neoadjuvant ADT for 4–6 months ± docetaxel, followed by EBRT and adjuvant ADT for 2 years. Based on the updated ESMO criteria, prostate cancer is classified as high-risk if a patient presents with a PSA level >20 ng/mL, a Gleason score ≥8 or a clinical stage ≥ T2c (6).

Management of metastatic prostate cancer is based on treatment intensification beyond ADT alone. In metastatic hormone-naive prostate cancer, ADT should be combined with either docetaxel or an androgen-receptor pathway inhibitor (ARPI) such as abiraterone, enzalutamide, or apalutamide, as these combinations significantly improve overall survival (OS). In selected patients with *de novo* low-volume metastatic disease, radiotherapy to the primary prostate tumor may further enhance outcomes. In metastatic castration-resistant prostate cancer (mCRPC), treatment consists of ARPIs, taxane-based chemotherapy (docetaxel or cabazitaxel if patient was previously treated with docetaxel), and other systemic options chosen on the basis of previous therapy, response duration, and clinical condition. Sequential use of similar ARPIs is discouraged due to limited efficacy (6).

Radioligand therapy has become an important and rapidly evolving treatment option in mCRPC, particularly after progression on first-line systemic therapies (5, 6). In view of the rapidly evolving role of PSMA-targeted radioligand therapy in mCRPC, this narrative review was undertaken to synthesize the available evidence on lutetium-177 (^177Lu)-based PSMA therapy and emerging actinium-225 (^225Ac)-based PSMA approaches, with particular attention to clinical efficacy, safety considerations, patient selection, therapeutic sequencing, and future directions.

## 2 Materials and methods

To collect all available data on the subject, we conducted a thorough search in the US National Library of Medicine (PubMed) and Google Scholar databases. The literature search was conducted between December 2025 and February 2026. The most recent papers were defined as those published within the past 6 years (from 2020 to 2026). However, given the limited data on specific historical aspects of the topic, older seminal research was also included.

To identify the most relevant papers, the search terms included the phrases: “radioisotope therapies” AND “prostate cancer”; “Actinium-225” AND “prostate cancer”; “Lutetium-177” AND “prostate cancer”; “alpha emitters” AND “prostate cancer”; “beta emitters” AND “prostate cancer”. While our search strategy included broad terms for alpha and beta emitters, the specific focus on Lutetium-177 and Actinium-225 was dictated by their distinct clinical relevance: Lutetium-177 has already received regulatory approval for clinical use, whereas Actinium-225 demonstrates highly promising efficacy data in ongoing clinical trials. In addition to peer-reviewed research articles and review papers, we analyzed the most important clinical guidelines on the management and treatment of prostate cancer (ESMO and NCCN), as well as regulatory documents issued by the FDA and EMA.

We included phase I–III clinical trials, retrospective cohort studies, meta-analyses, and systematic reviews. Crucial landmark studies (such as VISION and TheraP) were given priority during selection due to their transformative impact on clinical practice and current oncological guidelines. During the selection process, editorial articles, commentaries, letters to the editor without original data, and conference abstracts were excluded. Furthermore, articles written in a language other than English without an available translation, as well as publications that had been withdrawn or retracted, were excluded.

In order to comprehensively address the radionuclides currently used in the treatment of mCRPC, the review also included a dedicated section on radium-223, focusing on its established role, clinical indications, and relevance within the broader landscape of radionuclide therapy. Additionally, we used the ClinicalTrials.gov database to identify currently ongoing clinical trials using the search strings “PSMA radioligand therapy” and “prostate cancer”. The authors independently selected the studies, and any disagreements regarding the inclusion of studies or the interpretation of data were resolved through a consensus discussion.

A total of 782 entries were initially identified in the databases. After removing duplicates and reviewing the titles and abstracts against the eligibility criteria, 124 full-text articles were assessed. Ultimately, 58 publications were deemed eligible and included in this review.

## 3 Principles of radioligand therapy

Radioligands consist of three main components: a ligand, a radionuclide, and a linker between them. The role of the linker is not only to connect the radionuclide with the ligand, but also to ensure the stability of the radiopharmaceutical and the structural integrity of the entire unit. Each of the above-mentioned elements affects the efficacy, tumor selectivity and safety of the radiopharmaceutical. The appropriate selection and optimization of all elements is the most important aspect for the development of radioisotope therapies (7). Furthermore, the diversity of each of these elements allows for the design of various structures which, depending on the type of radiation emitted during the decay of the radioisotope, perform different functions. For this reason, we can divide them into diagnostic, therapeutic, and particles with common diagnostic and therapeutic properties (8).

Each radionuclide, as an unstable atom, releases energy in the form of ionizing radiation during decay. Generally, therapeutic radionuclides typically emit high-energy beta or alpha particles, which induce deoxyribonucleic acid (DNA) damage in cancer cells, potentially leading to cell death. In contrast, diagnostic radionuclides primarily emit gamma radiation, which penetrates tissue and can be detected by imaging devices such as gamma cameras or Positron Emission Tomography (PET) scanners. The gamma radiation emitted by diagnostic isotopes is of low energy and delivered in small doses, minimizing cellular damage and making these agents suitable for safe imaging of anatomical or functional processes. Thus, while both classes rely on the radioactive properties of isotopes, only therapeutic radionuclides exert a significant cytotoxic effect (7–9).

Table 2 below describes the types of radiation and their applications (7–9).

**TABLE 2:** Comparison of radiation emitted by radionuclides (7–9).

Type of radiation	α (alpha)	β^−^ (beta minus)	γ (gamma)	Auger
Tissue range	50–100 μm	mm-cm	Large	nm
Linear energy transfer (LET)	High	Low-medium	Very low	Locally very high
Typical application	Micrometastases, targeted cytotoxicity	Large tumors, crossfire effect	Therapy monitoring + imaging	Cell nucleus targeted cytotoxicity
Example nuclides	^225Ac, ^223Ra, ^213Bi	^177Lu, ^90Y	^131I	^125I, ^211 At

Legend: ^225Ac - Actinium-225; ^223Ra - Radium-223; ^213Bi - Bismuth-213; ^177Lu - Lutetium-177; ^90Y - Yttrium-90; ^131I - Iodine-131; ^125I - Iodine-125; ^211At - Astatine-211.

A ligand is a molecule that has the ability to bind to a specific antigen. In cancer therapies, it is used to recognize antigens on cancer cells or those found in the tumor microenvironment. The choice of ligand for radioligand therapy should be tailored to the biology of the tumor, the type and distribution of the antigen, and the individual characteristics of the patient. Ligands used in targeted therapy can be divided into three groups: antibodies, peptides, and small molecules. Each of them differs in their pharmacokinetic and pharmacodynamic properties (7, 9).

Monoclonal antibodies (mAbs) are characterized by a high degree of specificity towards antigens and a long circulation time in the blood. They allow for increased retention in the tumor and, at the same time, deliver more radiation. However, they must be used with caution in patients, as large amounts slow down elimination from non-targeted tissues (7, 9).

Small molecules are the easiest to synthesize and label with isotopes. They allow for high penetration into cancerous tissues and are quickly removed from the circulation, thus having a shorter residence time in the body. Unfortunately, this may result in insufficient radiation delivery to the tissues (7, 9).

Peptides, on the other hand, allow good tissue penetration while being eliminated faster than antibodies. They are often the best choice because they can be further chemically modified. This increases their stability and affinity for the antigen (7–9).

The linker plays a very important role in radioligand therapy. It is a molecule that connects the ligand with the radioisotope. In classic radioligand designs, the linker is passive, not susceptible to cleavage, but in radioligand therapy it has an active pharmacokinetic function. They allow for a reduction in the accumulation of radioactivity in the kidneys and improve the therapeutic index of the radioligand. Cleavable linkers are designed to release the radioligand fragment during elimination so that the radiometabolite is not retained in the kidneys but is rapidly excreted in the urine (10).

## 4 Radioligands in the treatment of prostate cancer

Radioligand therapy represents an important innovation in the treatment of advanced prostate cancer, particularly through targeting of the prostate-specific membrane antigen (PSMA) (11). PSMA, also known as glutamate carboxypeptidase e II, is physiologically expressed in several normal tissues, including prostatic epithelium, the small intestine, proximal renal tubules, and the salivary and lacrimal glands (12). In prostate cancer, PSMA expression is markedly upregulated, with levels reported to be 100–1,000 times higher than in benign prostate tissue (13). Expression further increases in metastatic and castration-resistant prostate cancer, underscoring its association with advanced disease. PSMA expression has also been documented in other malignancies, including renal cell carcinoma, hepatocellular carcinoma, and colorectal carcinoma. Notably, in prostate cancer, higher PSMA expression correlates with increasing Gleason score, suggesting an association with tumor aggressiveness (12, 14). The pronounced overexpression of PSMA in prostate cancer has established it as an attractive molecular target for radionuclide therapy. However, despite its nomenclature, PSMA is not exclusively prostate-specific, and its presence in normal tissues introduces a potential risk of off-target radiation exposure (11, 15).

### 4.1 PSMA-directed beta-emitters

Multiple classes of PSMA-directed radioligands labeled with ^177Lu have been developed for targeted radionuclide therapy in mCRPC. ^177Lu is a medium-energy β-emitting radionuclide with an average particle energy of approximately 133 keV and a maximum energy of about 0.5 MeV. Its β particles have limited tissue penetration, generally not exceeding 2 mm. This relatively short path length allows for highly targeted irradiation of small tumor lesions while effectively sparing surrounding healthy tissue (11, 16, 17). Crucially, this tissue range also enables the so-called “crossfire effect,” whereby neighboring tumor cells with low or heterogeneous PSMA expression may receive cytotoxic irradiation from radioligands bound to adjacent PSMA-expressing cells. This phenomenon may be advantageous in tumors with intralesional heterogeneity of PSMA expression. However, the β-particle range also introduces inherent limitations. In very small micrometastatic lesions, a proportion of the emitted radiation may be deposited outside the tumor volume, potentially reducing therapeutic efficacy. Similar limitations apply to lesions with globally low PSMA expression, which may fail to accumulate sufficient radiopharmaceutical activity to achieve an effective absorbed dose (18). Furthermore, the physical half-life of ^177Lu (approximately 6–7 days) presents a significant clinical advantage. It allows for the optimal delivery of the therapeutic radiation dose within a relatively short timeframe, thereby minimizing the risk of prolonged radiation exposure and associated adverse effects in healthy tissues (5, 16, 17).

**FIGURE 1:**
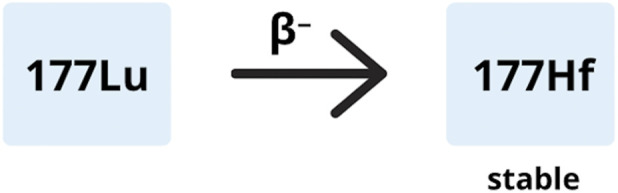
Disintegration of ^177Lu. Legend: ^177Lu - Lutetium-177; ^177Hf - Hafnium-177; β− - Beta-minus decay.

^177Lu compounds broadly fall into two categories: low-molecular weight PSMA inhibitors and PSMA-directed monoclonal antibodies. Clinical experience to date has predominantly involved small-molecule ligands due to their favorable pharmacokinetic and dosimetric characteristics (5, 11, 17).

The decay of ^177Lu is described in Figure 1.

Small-molecule PSMA radioligands are typically based on a urea-containing binding motif that mimics the natural substrate interaction within the catalytic pocket of PSMA. This pharmacophore is engineered to achieve high binding affinity and specificity, enabling selective accumulation in PSMA-overexpressing tumor tissue. The targeting unit is connected via a tailored linker to a macrocyclic chelator, which most commonly is Dodecane Tetraacetic Acid (DOTA) that forms a stable coordination complex with ^177Lu. The structural modularity of these agents allows optimization of tumor uptake, clearance kinetics, and radiation delivery. Following receptor engagement on the tumor cell surface, the ligand-receptor complex undergoes internalization, resulting in intracellular retention of the radionuclide. The emitted β particles induce cytotoxicity primarily through the formation of DNA double-strand breaks. Given the limited path length of β radiation in tissue, this approach permits localized energy deposition within tumor lesions while reducing irradiation of adjacent normal structures. Additionally, the gamma emissions of ^177Lu enable post-therapy imaging and dosimetry assessment (5, 16, 17).

In contrast, antibody-based PSMA radioconjugates display prolonged systemic circulation and higher blood-pool activity, which may translate into increased hematologic toxicity. Although antibodies offer strong target specificity, their larger molecular size can limit tumor penetration compared with small-molecule inhibitors. Collectively, the structural design of ^177Lu-labeled PSMA small molecules, comprising a high-affinity targeting motif, a pharmacokinetically optimized linker, and a robust chelator, underpins their better therapeutic efficacy and clinical utility in advanced prostate cancer (5, 11, 16).

Preclinical study evaluated the clinical application of small molecule inhibitors targeting PSMA: ^177Lu-DOTA-PSMA-617 (^177Lu-PSMA-617) and ^177Lu-DOTAGA-PSMA-I&T (^177Lu-PSMA-I&T), as well as the PSMA nanobody ^177Lu-DOTA-JVZ-007 (^177Lu-JVZ-007), using PSMA-expressing cell lines, a unique set of prostate cancer patient tumors and healthy human tissue. The results showed that ^177Lu-PSMA-617 has better *in vitro* binding properties on PSMA-expressing cells than ^177Lu-PSMA-I&T, as well as ^177Lu-JVZ-007, Importantly ^177Lu-PSMA-617 had lower affinity to the kidney and salivary gland tissue samples compared to ^177Lu-PSMA-I&T and ^177Lu-JVZ-007. The above preclinical evaluation indicates the potential of the ^177Lu-PSMA-617 in the context of PSMA-targeted radioisotope therapy, with particular emphasis on the increased safety of the treatment, as it has low binding affinity to other organs expressing PSMA, such as the small intestine, the central nervous system, proximal renal tubules and the lacrimal and salivary glands. This is also important in the context of quality of life of the patients, because the potential toxicity to these organs, especially the salivary glands, may significantly affect patients' quality of life (19).

The phase III VISION clinical trial evaluated the efficacy and safety of ^177Lu-PSMA-617 in patients with mCRPC who had positive PSMA PET/CT scan results. These patients had previously been treated with ADT and taxanes and had to have PSMA-positive status determined with the use of centrally read gallium-68 (^68Ga)-labeled PSMA-11 (^68Ga-PSMA-11) PSMA PET/CT imaging at baseline. A total of 831 patients were randomized in 2:1 ratio to the ^177Lu-PSMA-617 group and control group. Patients in the study group received 7.4 GBq of ^177Lu-PSMA-617 administered intravenously every 6 weeks for 4-6 cycles combined with standard care. Participants in control group received s2tandard care alone, which excluded chemotherapy, immunotherapy, Radium-223 (^223Ra) and experimental treatment. The results showed that ^177Lu-PSMA-617 significantly prolonged median progression-free survival (PFS) as compared to the control group, with median PFS of 8.7 months vs. 3.4 months respectively [hazard ratio (HR), 0.40; 99.2% confidence interval (CI), 0.29 to 0.57; P < 0.001). Similarly, median OS was also beneficial in the study group when compared to the control group (15.3 vs. 11.3 months respectively, HR 0.62; 95% CI, 0.52 to 0.74; P < 0.001). The therapy was also superior in secondary endpoints such as objective response, disease control and time to skeletal events. The most common adverse events included fatigue in 228 patients (43.1%), dry mouth in 205 patients (38.8%), and nausea in 187 patients (35.3%). In the VISION cohort, adverse events of grade 3 or higher were observed in approximately 52.7% of patients treated with ^177Lu-PSMA-617, compared with 38.0% in the control arm, reflecting an expected increase in treatment-related toxicity due to targeted radiation exposure. Haematological toxicity was the most important element of the safety profile, with grade ≥3 anemia occurring in about 12.9%, thrombocytopenia in 7.9%, and leukopenia in 7.8% of patients in the study group. Other non-hematologic grade ≥3 events were less common, including back pain (3.2%), arthralgia (1.1%), decreased appetite (1.9%), constipation (1.1%), diarrhea (0.8%), and vomiting (0.9%). No significant renal toxicities of grade ≥3 were reported in dosimetry substudy analyses, and hepatic toxicities were rare and generally low grade, supporting a favorable organ-specific safety profile for ^177Lu-PSMA-617 therapy. These data underscore that bone marrow suppression remains the primary dose-limiting toxicity of ^177Lu-PSMA-617. Dose modifications were necessary in a subset of patients: 30 patients (5.7%) required at least one dose reduction, 85 patients (16.1%) had temporary treatment interruptions, and 63 patients (11.9%) discontinued ^177Lu-PSMA-617 due to adverse events. Deaths attributed to adverse events occurred in 19 patients (3.6%), however it should be noted that investigators attributed five fatal adverse events in the ^177Lu-PSMA-617 group to the therapy, including pancytopenia in two patients, bone marrow failure in one patient, subdural hematoma in one patient, and intracranial hemorrhage in one patient (20).

In the phase II randomized clinical trial TheraP, 200 patients with mCRPC and PSMA-positive metastatic disease who were considered suitable for cabazitaxel chemotherapy were randomly assigned in a 1:1 ratio to receive either ^177Lu-PSMA-617 radioligand therapy or cabazitaxel. Patients allocated to the experimental arm received up to six cycles of ^177Lu-PSMA-617 administered every 6 weeks, starting at 8.5 GBq for cycle 1 with planned de-escalation by 0.5 GBq per subsequent cycle. Patients in the control arm received cabazitaxel at a dose of 20 mg/m^2^ intravenously every 3 weeks for up to ten cycles. The primary endpoint was a PSA response defined as a ≥50% decline from baseline. A PSA reduction of at least 50% was achieved in 66% of patients treated with ^177Lu-PSMA-617 compared with 37% in the cabazitaxel group (difference of 29%; p < 0.0001), demonstrating superior biochemical response with radioligand therapy. Objective response rates and PFS also favored the ^177Lu-PSMA-617 arm. Treatment was generally better tolerated in the radioligand group. Grade 3–4 adverse events occurred in 33% of patients receiving ^177Lu-PSMA-617 versus 53% in the cabazitaxel arm. Hematologic toxicity was more frequent with cabazitaxel, whereas xerostomia was more commonly observed with radioligand therapy. No treatment-related deaths were attributed to ^177Lu-PSMA-617 (21).

The VISION and TheraP studies have been compared in Table 3.

**TABLE 3:** Comparison of VISION and TheraP studies (20,21).

Compared elements	VISION study	TheraP study
Research phase	Phase III, randomised, open-label, multicentre	Phase II, randomised, open-label, multicentre
Number of participants	831 patients	200 patients
Inclusion criteria	Progressive mCRPC after 2 lines of therapy including ADT and taxanes, with positive metastases in PSMA	Progressive mCRPC with PSMA-positive metastases
The factor under investigation	^177Lu-PSMA-617 + standard care vs. standard care, 2:1	^177Lu-PSMA-617 vs. cabazitaxel, 1:1 randomization
Treatment plan	^177Lu-PSMA-617 at a dose of 7.4 GBq every 6 weeks, up to a maximum of 6 cycles	^177Lu-PSMA-617 every 6 weeks, maximum 6 cycles (starting at 8.5 GBq, then decreasing to 6.0 GBq) vs cabazitaxel
Summary	^177Lu-PSMA-617 and SOC therapy significantly prolongs OS and PFS in patients with PSMA-positive mCRPC after prior treatment	^177Lu-PSMA-617 is a good alternative to cabazitaxel in PSMA-positive mCRPC. It is characterised by a higher response rate and a better safety profile, but without a significant prolongation

Legend: mCRPC, metastatic Castration-Resistant Prostate Cancer; ADT -Androgen Deprivation Therapy; PSMA, Prostate-Specific Membrane Antigen; ^177Lu–Lutetium-177; GBq, GigaBecquerel; SOC, Standard Of Care; OS, Overall Survival; PFS, Progression-Free Survival.

The phase III PSMAfore trial was designed to evaluate the efficacy and safety of ^177Lu-PSMA-617 in patients with PSMA-expressing taxane-naive mCRPC who had experienced disease progression on at least one prior ARPI. A total of 585 patients were screened, of whom 468 fulfilled eligibility criteria and were randomly assigned in a 1:1 ratio to receive either ^177Lu-PSMA-617 or a different ARPI. Patients assigned to the experimental arm received up to six cycles of intravenous ^177Lu-PSMA-617 administered every 6 weeks. Patients in the control arm received an alternative ARPI. Primary endpoint was radiographic progression-free survival (rPFS), assessed by blinded independent central review. In the primary analysis, median rPFS was 9.3 months (95% CI 6.77-not estimable) in the ^177Lu-PSMA-617 group compared with 5.6 months (95% CI 4.04–5.95) in the ARPI group. With extended follow-up of 24 months, median rPFS increased to 11.6 months (95% CI 9.3–14.2) in the radioligand arm, whereas it remained approximately 5.6 months (95% CI 4.2–6.0) in the control group, confirming a sustained and clinically meaningful delay in radiographic progression. These findings support the use of PSMA-targeted radioligand therapy earlier in the disease course, prior to exposure to taxane-based chemotherapy. In the initial OS analysis, the difference between groups did not reach statistical significance (24.5 months for ^177Lu-PSMA-617 versus 23.2 months for ARPI). This result was largely influenced by a substantial crossover rate, as many patients in the control arm subsequently received radioligand therapy after progression. Adjusted analyses accounting for crossover suggested an OS benefit favoring ^177Lu-PSMA-617, reinforcing the clinical relevance of the observed rPFS improvement. ^177Lu-PSMA-617 demonstrated a manageable and predictable toxicity profile consistent with prior studies such as VISION and TheraP. The most frequently observed adverse events were hematologic, including anemia, thrombocytopenia, and leukopenia, reflecting the bone marrow irradiation associated with systemic radioligand therapy. Most hematologic toxicities were low to moderate in severity and manageable with standard treatment. Non-hematologic adverse events commonly included fatigue, nausea, and xerostomia. Importantly, severe treatment-related adverse events occurred at rates considered acceptable in the context of advanced mCRPC, and the overall tolerability profile compared favorably with that of cytotoxic chemotherapy. Treatment discontinuations due to adverse events were relatively rare, and no unexpected safety signals emerged (22).

### 4.2 Current role of alpha-emitting Radium-223

Radium-223 (^223Ra) is an alpha-emitting radiopharmaceutical that selectively targets areas of increased bone turnover, delivering high-energy radiation with a short tissue range. Owing to its chemical similarity to calcium, ^223Ra selectively localizes to areas of increased osteoblastic activity, where it becomes incorporated into the bone matrix adjacent to metastatic lesions. ^223Ra that is not incorporated into bone is rapidly cleared, predominantly via the gastrointestinal tract (23–25).

Its clinical efficacy was established in the phase III ALSYMPCA trial, which enrolled 921 patients with progressive, bone-predominant, symptomatic mCRPC, who had at least two bone metastases and no known visceral metastases. Patients were randomized in a 2:1 ratio to receive six intravenous injections of ^223Ra (n = 614) or placebo (n = 307), both in combination with best supportive care. ^223Ra significantly prolonged OS, with a median OS of 14.9 months versus 11.3 months in the placebo group (HR 0.70; 95% CI 0.58–0.83; P < 0.001), and delayed the time to the first symptomatic skeletal event, with a median of 15.6 months compared with 9.8 months for placebo (HR 0.66; 95% CI 0.52–0.83; P < 0.001). Treatment was generally well tolerated, with low rates of grade 3 hematologic toxicity including thrombocytopenia (in 3% of the study group) and diarrhoea (in 2% of the study group), reflecting the limited penetration of alpha particles into surrounding tissues (26). However, subsequent studies prompted important safety considerations. The ERA-223 trial demonstrated a substantially increased incidence of fractures when ^223Ra was administered in combination with abiraterone-prednisone combination (28.6% vs. 11.4%), without evidence of clinical benefit. Therefore, co-administration of ^223Ra with abiraterone and prednisone/prednisolone is contraindicated (27). ^223Ra remains a valuable later-line option for patients with symptomatic bone-dominant metastatic castrate-resistant prostate cancer without visceral metastases, particularly those with pain or skeletal morbidity where disease control and quality-of-life (QoL) improvement are primary goals. Its use should be limited to patients who have received at least two prior systemic therapies for metastatic castrate-resistant prostate cancer or are ineligible for these treatments (6).

### 4.3 PSMA-directed alpha-emitters

Therapy using ^225Ac is based on alpha radiation, i.e., the emission of helium nuclei. Alpha particles are characterized by very low tissue penetration (approximately 50–100 μm) and high linear energy transfer (LET) of about 80 keV/μm, which results in highly localized and potent cytotoxicity. Owing to this short range, ^225Ac therapy enables selective tumor cell killing while reducing radiation exposure to surrounding non-malignant tissues compared with beta-emitting radionuclides (28–30).

^225Ac-PSMA therapy is primarily intended for patients with mCRPC, often after failure of multiple prior conventional treatment modalities (31). Importantly, unlike ^223Ra, which is indicated only for bone metastases without visceral involvement, ^225Ac-PSMA radioligand therapy can target metastatic lesions in the bones as well as in visceral organs such as the lungs and liver, and potentially in the brain (31–33). Furthermore, clinical investigations are ongoing to evaluate its use in patients who have progressed after ^177Lu-PSMA-617 therapy. Due to the higher LET and distinct radiobiological effects of alpha particles, ^225Ac may overcome resistance to beta-emitting radioligands in selected cases. Emerging data suggest that earlier introduction of ^225Ac-PSMA therapy or its combination with other systemic treatments may enhance therapeutic outcomes (34–36).

The therapeutic effect of ^225Ac results from its decay cascade, during which it emits four alpha particles along with accompanying beta and gamma emissions in multiple decay steps before reaching stable ^209Bi (29, 37).

**FIGURE 2:**
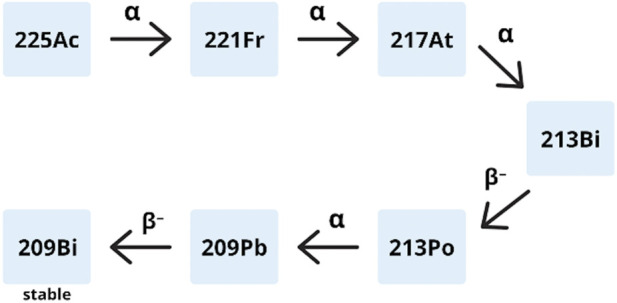
Disintegration of ^225Ac. Legend: ^225Ac–Actinium-225; ^221Fr - Francium-221; ^217At - Astatine-217; ^213Bi - Bismuth-213; ^213Po - Polonium-213; ^209Pb - Lead-209; ^209Bi - Bismuth-209; α - Alpha decay; β− - Beta-minus decay.

^225Ac has a physical half-life of 9.9 days, which allows for sustained tumor irradiation following administration (29, 30). The total energy released throughout its decay chain is approximately 28 MeV. During decay, several daughter radionuclides are generated. Redistribution of these daughter isotopes may contribute to off-target accumulation in organs such as the kidneys, liver, spleen, lungs, intestines, and bone marrow, potentially leading to organ toxicity (31, 37).

Disintegration of ^225Ac is presented on Figure 2.

To improve *in vivo* stability, ^225Ac is complexed with bifunctional chelators such as DOTA, DOTATOC, DO3A, PEPA, HEHA, TETA, EDTA, CHX-DTPA, and others (37, 38). These chelators do not remove radioactive residues from the body, rather forming stable coordination complexes with the radionuclide, thereby minimizing premature release and reducing nonspecific organ uptake. Low-intensity gamma emissions from the decay chain may additionally enable post-therapy imaging and dosimetric assessment using Single-Photon Emission Computed Tomography (SPECT) (31).

^225Ac is most commonly conjugated to small-molecule PSMA ligands such as PSMA-617 or PSMA-I&T, which demonstrate favorable pharmacokinetics and improved tumor penetration compared with monoclonal antibodies (37–39). An alternative strategy involves labeling the anti-PSMA monoclonal antibody J591 with ^225Ac. Antibody-based constructs may provide prolonged tumor retention but are associated with increased hematologic toxicity due to longer systemic circulation (39).

Currently the ^225Ac conjugates are not approved in the treatment of prostate cancer. Several phase I and II clinical trials (e.g., NCT03276572, NCT04576871, NCT04886986, NCT04946370, NCT04506567) are currently investigating ^225Ac-J591 to determine optimal dosing, cumulative maximum tolerated dose (MTD), dose-limiting toxicities (DLTs), and potential efficacy both as monotherapy and in combination with ^177Lu-PSMA-I&T, pembrolizumab, radiotherapy, or androgen deprivation therapy (38, 39). Other PSMA-targeted ^225Ac conjugates are currently under investigation including ^225Ac complexes with PSMA-617, PSMA-I&T, TLX592, RPS-074, SibuDAB, h11B6 antibody, and PNT2001 (38–40).

Selected ongoing trials are summarized in Table 4.

**TABLE 4:** Chosen ongoing clinical trials of ^225Ac-J591 in treatment of mCRPC.

Study name	NCT number	Disease setting	Investigated factors
Phase I dose-escalation trial of ^225Ac-J591 in patients with mCRPC	NCT03276572	mCRPC	MTD, DLT, PSA response, CTC
Re-treatment ^225Ac-J591for mCRPC (pilot study)	NCT04576871	mCRPC	Safety of the treatment and adverse event profile
Phase I/II ^225Ac-J591 plus ^177lu-psma-i&T for progressive mCRPC	NCT04886986	Progressive mCRPC	Combination of radio-ligand therapy, DLT, MTD, PSA decline and radiology response
Phase I/II trial of pembrolizumab + AR inhibitor with or without ^225Ac-J591 for progressive mCRPC	NCT04946370	Progressive mCRPC	Safety, dose and efficiency of combination of immunotherapy with androgen receptor pathway inhibitor with^225^Ac
Phase I/II dose-escalation study of fractionated and multiple dose ^225Ac-J591 for progressive mCRPC	NCT04506567	Progressive mCRPC	MTD, DLT, optimal dosing schedule

Legend: NCT - National Clinical Trial; ^225Ac - Actinium-225; mCRPC–metastatic Castrate-Resistant Prostate Cancer; MTD - Maximum Tolerated Dose; DLT - Dose-Limiting Toxicity; PSA - Prostate-Specific Antigen; CTC - Circulating Tumor Cells; ^177Lu - Lutetium-177; AR - Androgen Receptor.

With increasing clinical experience, based mostly on the retrospective studies of ^225Ac-PSMA-617 and ^225Ac-PSMA-I&T conjugates, more data on this form of therapy is becoming available. Meta-analyses indicate that PSA decline following ^225Ac-PSMA therapy occurs in approximately 70%–80% of patients, with a ≥50% reduction observed in more than half of treated individuals (34, 36, 41). Improved responses have been reported in patients who were not previously treated with other radioligand therapies such as ^177Lu-PSMA-617 (33, 36). Median PFS is approximately 8–10 months, while median OS ranges from 12 to 18 months, with selected patients achieving prolonged survival extending to several years (33–36, 41).

The most commonly reported adverse effects of ^225Ac therapy are xerostomia and hematologic toxicity, including anemia, leukopenia, and thrombocytopenia (33, 36, 39). Xerostomia results from physiological PSMA expression in salivary glands and represents the principal dose-limiting toxicity. Various mitigation strategies, including external cooling, botulinum toxin injections, competitive PSMA inhibitors (e.g., polyglutamate-based compounds), glucocorticosteroids, and anticholinergic agents, have been investigated. However, their efficacy remains limited and inconsistent (33, 39). Grade 3–4 hematologic toxicities occur less frequently but are clinically significant, particularly in heavily pretreated patients or those with extensive bone marrow involvement (34, 36). Nephrotoxicity of varying severity (grades 1–4) has also been reported and may be related to renal PSMA expression and redistribution of radioactive daughter nuclides (37, 42). Less common adverse effects that may be attributable to therapy rather than disease progression include nausea, vomiting, anorexia, weight loss, fatigue, constipation, hypoalbuminemia, dysuria, and dry eye syndrome (33, 36, 39).

### 4.4 Toxicity, dosimetry, and quality-of-life considerations

Bone marrow suppression represents the primary dose-limiting toxicity for ^177Lu-PSMA-617. In the phase III VISION trial, grade ≥3 anemia occurred in 12.9% of patients, alongside thrombocytopenia (7.9%) and leukopenia (7.8%) (20). Hematologic toxicity is also a significant concern with ^225Ac-PSMA, particularly in patients who have been heavily pretreated with cytotoxic chemotherapy or have extensive bone marrow metastatic involvement (43). Based on the meta-analysis of ^225Ac-PSMA studies, grade ≥3 anemia was the most frequently reported grade ≥3 toxicity, occurring in 20 out of 186 patients (10.75%) across seven studies. Other hematologic toxicities were evaluated in six studies, pooling a total of 169 patients and among these, leukopenia was observed in 10 patients (5.9%), while thrombocytopenia was recorded in 8 patients (4.73%) (34). Depletion of the marrow reserve over consecutive cycles requires vigilant monitoring of complete blood counts prior to each administration for both therapies.

Physiological PSMA expression within the salivary glands results in significant off-target radiation exposure. In the landmark phase III VISION trial, xerostomia was reported in 38.8% of patients treated with ^177Lu-PSMA-617, though these events were predominantly mild (grade 1–2) and clinically manageable (20). Conversely, xerostomia occurs substantially more frequently during ^225Ac-PSMA therapy, representing its primary dose-limiting toxicity. The high LET of alpha particles induces profound, irreversible damage to the salivary gland acinar cells, resulting in severe dry mouth that frequently restricts the maximum tolerated dose or the frequency of radioligand administration (43, 44). In the meta-analysis conducted by Parida et al., xerostomia was confirmed as the most prevalent adverse event associated with ^225Ac-PSMA, affecting 167 out of 226 pooled patients (73.9%). The vast majority of these cases were low-grade (grades I–II), whereas high-grade toxicity was exceedingly rare, with grade ≥3 xerostomia documented in only a single patient across cohorts utilizing standardized grading systems (34).

Similar to the salivary glands, the lacrimal glands exhibit physiological PSMA expression, leading to unintended off-target radiation exposure and subsequent xerophthalmia (dry eye syndrome). In the phase III VISION trial, dry eye was reported in 3% of patients treated with ^177Lu-PSMA-617, with the vast majority of cases being mild (grade 1–2) and clinically manageable (20). Lacrimal gland toxicity during 225Ac-PSMA therapy is driven by the high LET of alpha particles causing direct damage to the lacrimal acinar cells. Notably, this specific adverse event was frequently underreported or omitted in early landmark alpha-therapy cohorts (34).

Renal toxicity appears relatively uncommon in both therapies when appropriate patient selection is applied. In the VISION trial, renal adverse events of any grade occurred in approximately 9% of patients, with grade ≥3 events in roughly 3%–4%, and no clear signal of clinically significant irreversible nephrotoxicity was observed (20). For ^225Ac-PSMA, renal toxicity is less well defined due to smaller and more heterogeneous cohorts. However, nephrotoxicity associated with ^225Ac-PSMA appears to be uncommon and is generally reported in a minority of patients, with pooled estimates suggesting an incidence of approximately 3%–4% across available studies, most of which are low-grade events (34). The interpretation of renal safety in alpha-emitting radioligand therapy remains challenging due to heterogeneous reporting and limited standardized dosimetric assessment. Theoretical concerns exist regarding renal radiation exposure, primarily related to the decay cascade of ^225Ac and the recoil effect, which may result in partial detachment of daughter radionuclides (such as ^213Bi) from the targeting vector and their subsequent redistribution, including potential accumulation in non-target organs such as the renal cortex (30, 45). However, current clinical evidence does not consistently demonstrate a significant or dose-limiting nephrotoxicity signal in treated cohorts (34).

Fatigue and gastrointestinal symptoms are among the most frequently reported non-hematologic adverse events in both treatment modalities. In the VISION trial, fatigue occurred in 43.1% of patients receiving ^177Lu-PSMA-617, while nausea, decreased appetite, and mild gastrointestinal discomfort were also common but generally low grade (20). In ^225Ac-PSMA cohorts, fatigue has been reported in around 45% of patients, although most events are grade 1–2 and are likely multifactorial, reflecting both treatment-related effects and advanced disease burden (45).

Cumulative toxicity represents an important clinical consideration for both ^177Lu-PSMA-617 and ^225Ac-PSMA, particularly because radioligand therapy is usually administered over repeated treatment cycles. In the VISION trial, adverse events led to ^177Lu-PSMA-617 dose reduction 5.7% patients, treatment interruption in 16.1% patients, and permanent discontinuation in 11.9% patients. The publication does not provide a detailed breakdown of all individual adverse events responsible for these treatment modifications. However, the severe toxicity profile was dominated by hematologic events (20).

For ^225Ac-PSMA, directly comparable data on dose reduction and treatment interruption are not consistently reported across retrospective cohorts, which limits formal comparison with VISION. Nevertheless, available studies indicate that treatment-limiting toxicity is driven predominantly by cumulative salivary gland toxicity, with hematologic toxicity remaining clinically relevant in heavily pretreated patients. In the post-^177Lu-PSMA-617 salvage cohort reported by Feuerecker et al., a median of 2 cycles of ^225Ac-PSMA were administered to 26 patients, and treatment was stopped because of hematologic toxicity in 2 patients (7.7%) and due to xerostomia in 6 patients (23.1%) (43). In the larger WARMTH Act multicenter retrospective cohort of 488 patients who received ^225Ac-PSMA, with a median of two cycles per patient, xerostomia was reported after the first cycle in 236 of 347 evaluable patients (68%), and all patients who received more than seven cycles reported xerostomia, supporting the cumulative nature of salivary gland toxicity (46). In the same WARMTH Act cohort, grade ≥3 anemia, leukopenia, thrombocytopenia, and renal toxicity occurred in 13%, 4%, 7%, and 5% of patients, respectively, with no serious adverse events or treatment-related deaths recorded (46). Collectively, these data suggest that cumulative treatment limitation differs between modalities: for ^177Lu-PSMA-617, clinically relevant treatment modification is most closely linked to marrow suppression, whereas for ^225Ac-PSMA, xerostomia represents the dominant cumulative and treatment-limiting adverse effect, with hematologic toxicity remaining important in salvage populations with compromised marrow reserve.

Cumulative radiation exposure is an important but still incompletely standardized aspect of PSMA-targeted radioligand therapy. For ^177Lu-PSMA-617, repeated administration results in cumulative absorbed doses to dose-limiting organs, particularly the bone marrow, kidneys, salivary glands, and lacrimal glands. In VISION, ^177Lu-PSMA-617 was administered at 7.4 GBq every 6 weeks for up to six cycles, illustrating the importance of repeated-cycle exposure in both efficacy and toxicity assessment (20) Although beta-emitter dosimetry is more established than alpha-emitter dosimetry, routine individualized dosimetry is not universally implemented in clinical practice, and treatment decisions are still frequently guided by fixed activity schedules combined with clinical and laboratory monitoring. For ^225Ac-PSMA, cumulative radiation exposure is even more difficult to quantify because alpha-particle dosimetry is technically challenging, highly dependent on microdistribution, and complicated by the decay cascade of ^225Ac and possible redistribution of daughter radionuclides (30, 45). As a result, cumulative toxicity assessment for ^225Ac-PSMA relies not only on administered activity but also on clinical indicators such as worsening xerostomia, cytopenias, renal function changes, and treatment tolerance across cycles.

Patient-reported outcomes (PROs) are particularly relevant in PSMA radioligand therapy because several adverse events, especially xerostomia, fatigue, pain, and functional decline, may be underestimated by conventional Common Terminology Criteria for Adverse Events (CTCAE) grading. In the VISION Health-Related Quality of Life (HRQoL) analysis, ^177Lu-PSMA-617 plus standard of care delayed time to worsening in FACT-P total score, BPI-SF pain intensity, and EQ-5D-5L utility score compared with standard of care alone, indicating that the survival benefit of ^177Lu-PSMA-617 was accompanied by preservation of patient-reported quality of life and pain control (20). However, comparable prospective PROs data are largely lacking for ^225Ac-PSMA. Most available alpha-therapy studies focus on biochemical response, imaging response, survival, and clinician-reported toxicity, whereas standardized PROs instruments are inconsistently used. This represents an important limitation, because xerostomia caused by ^225Ac-PSMA is often classified as grade 1–2 but may substantially impair swallowing, speech, taste, sleep, oral hygiene, and nutritional intake. Therefore, future trials should incorporate validated PROs tools, including xerostomia-specific questionnaires, fatigue scales, pain inventories, and global quality-of-life instruments, to better capture the true patient-perceived burden of treatment (34, 43, 45–47).

Practical mitigation strategies for PSMA-targeted radioligand therapy remain only partly standardized. For ^177Lu-PSMA-617, the most established measures are careful patient selection, baseline assessment of hematologic, renal, and hepatic function, hydration when clinically appropriate, serial monitoring of complete blood counts and renal parameters, and treatment delay or discontinuation in the setting of clinically relevant toxicity (15, 16, 20). For ^225Ac-PSMA, toxicity mitigation is more challenging because salivary gland toxicity is common, cumulative, and difficult to prevent. In clinical practice and published retrospective cohorts, the most consistently applied strategies are individualized activity selection, prolongation of inter-cycle intervals, dose reduction, and treatment discontinuation when xerostomia or cytopenias become clinically limiting (34, 43, 46). Tandem approaches combining lower-activity ^225Ac-PSMA with ^177Lu-PSMA-617 have also been explored as a potential method to reduce salivary gland toxicity while preserving antitumor efficacy, although the evidence remains heterogeneous and non-randomized (47). More specific salivary gland protection strategies, including external cooling, sialendoscopy with saline irrigation and steroid injection, botulinum toxin injection, pilocarpine, amifostine, and other sialoprotective measures, have been investigated mainly in small series, proof-of-concept reports, or broader supportive-care contexts rather than as validated components of standardized ^225Ac-PSMA protocols (48–50). Therefore, these approaches should be regarded as experimental or institution-dependent, and their ability to prevent irreversible radiation-induced xerostomia remains uncertain. At present, mitigation of ^225Ac-PSMA toxicity relies primarily on prevention through careful patient selection, early symptom recognition, individualized dosing, treatment spacing, and timely discontinuation rather than on reliably effective organ-protective interventions.

While both ^177Lu-PSMA-617 and ^225Ac-PSMA target the same molecular receptor, the distinct physical properties of beta and alpha radiation lead to significantly different toxicity profiles. A comprehensive understanding of these adverse events is essential for multidisciplinary teams to optimize patient outcomes and maintain QoL. The primary differences in toxicity profiles between these two modalities are summarized in Table 5.

**TABLE 5:** Comparative toxicity profile of ^177Lu-PSMA and ^225Ac-PSMA radioligand therapy.

Toxicity domain	^177Lu-PSMA	^225Ac-PSMA	Main clinical implication
Radiation characteristics	Beta emitter with relatively lower LET and longer tissue penetration range	Alpha emitter with very high LET and short path length. Toxicity assessment is complicated by microdistribution and the ^225Ac decay cascade	Different radiation properties result in distinct toxicity patterns despite targeting the same PSMA receptor
Hematologic toxicity/marrow reserve	In VISION, grade 3 anemia occurred in 12.9%, thrombocytopenia in 7.9%, and leukopenia in 7.8% of patients (20)	In the meta-analysis by parida et al., grade ≥3 anemia occurred in 10.75% patients, leukopenia in 5.9% patients and thrombocytopenia in 4.73% patients (34). In WARMTH Act, grade ≥3 anemia, leukopenia, and thrombocytopenia occurred in 13%, 4%, and 7%, respectively (46)	Marrow reserve is clinically relevant for both modalities. For ^177Lu-PSMA, hematologic toxicity is a major treatment-limiting concern, whereas for ^225Ac-PSMA, risk is particularly relevant in heavily pretreated patients and those with extensive skeletal involvement
Xerostomia/salivary gland toxicity	In VISION, xerostomia occurred in 38.8% of patients, predominantly grade 1–2 and clinically manageable (20)	In parida et al., xerostomia occurred in 73.9% patients, with grade ≥3 xerostomia reported in only one patient across cohorts using standardized grading (34). In WARMTH Act, xerostomia was reported after the first cycle in 68% evaluable patients and all patients receiving more than seven cycles reported xerostomia (46)	Xerostomia is substantially more frequent with ^225Ac-PSMA and represents the dominant cumulative and treatment-limiting toxicity, despite usually being low grade by CTCAE criteria
Lacrimal gland toxicity/dry eye syndrome	In VISION, dry eye was reported in 3% of patients and was mostly mild (20)	Lacrimal gland toxicity is biologically plausible due to physiological PSMA expression, but it has been inconsistently reported and likely underrecognized in early alpha-therapy cohorts (34)	The true incidence of dry eye after ^225Ac-PSMA is uncertain because systematic ophthalmologic assessment is rarely incorporated into clinical studies. Further, phase III studies are needed
Renal toxicity and dosimetry	In VISION, renal adverse events of any grade occurred in approximately 9%, with grade ≥3 renal events in roughly 3%–4% (20)	Pooled estimates suggest nephrotoxicity in approximately 3%–4% of patients, mostly low grade (34). In WARMTH Act, grade ≥3 renal toxicity occurred in 5% (46). Theoretical concerns relate to ^225Ac daughter radionuclide redistribution and possible renal cortical accumulation (30, 45)	Clinically significant nephrotoxicity appears uncommon for both therapies, but renal dosimetry is more difficult to standardize for alpha emitters
Fatigue and gastrointestinal symptoms	In VISION, fatigue occurred in 43.1% of patients receiving ^177Lu-PSMA. Nausea, decreased appetite, and mild gastrointestinal symptoms were also common but generally low grade (20)	In ^225Ac-PSMA cohorts, fatigue has been reported in around 45% of patients, mostly grade 1–2, although symptoms are likely multifactorial and influenced by advanced disease burden (43)	Fatigue and gastrointestinal symptoms are common but usually low grade. Attribution to treatment alone is difficult in heavily pretreated mCRPC populations
Treatment modification and discontinuation	In VISION, adverse events led to dose reduction in 5.7%, treatment interruption in 16.1%, and permanent discontinuation in 11.9% of patients. The severe toxicity profile was dominated by hematologic events (20)	Comparable dose reduction and interruption data are not consistently reported across ^225Ac-PSMA retrospective cohorts. In feuerecker et al., treatment was stopped due to hematologic toxicity in 7.7% patients and due to xerostomia in 23.1% patients (43)	For ^177Lu-PSMA, treatment modification is most closely linked to marrow suppression, whereas for ^225Ac-PSMA, cumulative xerostomia is the main treatment-limiting toxicity
Patient-reported outcomes (PROs)	In the VISION HRQoL analysis, ^177Lu-PSMA plus standard of care delayed worsening in FACT-P, BPI-SF pain intensity, and EQ-5d-5L utility score compared with standard of care alone	Prospective PRO data are largely lacking for ^225Ac-PSMA. Most studies focus on PSA response, imaging response, survival, and clinician-reported toxicity	PROs are especially important for toxicities such as xerostomia, fatigue, pain, and functional decline, which may be underestimated by CTCAE grading
Overall toxicity pattern	More strongly associated with clinically relevant hematologic toxicity and treatment modification due to marrow suppression	More strongly associated with salivary gland toxicity, especially cumulative xerostomia, while hematologic toxicity remains relevant in salvage populations	^177Lu-PSMA and ^225Ac-PSMA require different toxicity-monitoring priorities despite sharing the same molecular target.

Legend: CTCAE, common terminology criteria for adverse events; HRQoL, health-related quality of life; LET, linear energy transfer; PROs, patient-reported outcomes; ^225Ac - Actinium-225; ^177Lu - Lutetium.

## 5 Discussion

Radioligand therapy has fundamentally transformed the management of advanced prostate cancer, particularly in patients with mCRPC, where therapeutic options become progressively limited and prognosis remains unfavorable. Earlier systemic radionuclide approaches, including bone-seeking β-emitters such as ^89Sr and ^153Sm, were primarily introduced as palliative modalities aimed at alleviating skeletal-related symptoms in patients with diffuse bone metastases. Although these agents provided clinically meaningful pain relief, they were associated with relevant myelosuppression and did not demonstrate a significant OS benefit; therefore, they did not alter the natural history of the disease (51–55). The transition from non-specific bone-targeted radionuclides to molecularly directed radioligands represents a major paradigm shift, aligning nuclear medicine with the principles of precision oncology. The identification of PSMA as a theranostic target has been central to this transformation. The integration of PSMA PET/CT imaging for patient selection before therapy has further enhanced treatment personalization by confirming target expression, improving the likelihood of therapeutic benefit, and limiting unnecessary toxicity (11–15).

Among β-emitting radioligands, ^177Lu-PSMA-617 is the most extensively studied and clinically validated compound. Randomized clinical trials, including VISION and TheraP, demonstrated significant improvements in rPFS, OS, and biochemical response rates compared with standard-of-care treatments in patients with advanced mCRPC (20, 21). These studies established ^177Lu-PSMA-617 as a survival-prolonging therapy rather than merely a palliative intervention. ^177Lu-PSMA-617 therapy is generally well tolerated, with the majority of patients completing multiple cycles. Hematologic toxicities are the most frequent severe adverse events, and a minority of patients require dose adjustments, temporary interruptions, or permanent discontinuation, highlighting the importance of careful monitoring and supportive care during therapy. The relatively short tissue penetration of β-particles, approximately 1–2 mm, allows for a crossfire effect sufficient to irradiate heterogeneous tumor cell populations while sparing surrounding normal structures (20, 21). The PSMAfore trial further expanded the therapeutic role of ^177Lu-PSMA-617 b y demonstrating a significant rPFS benefit in taxane-naïve patients, supporting its integration earlier in the treatment algorithm (22).

However, VISION, TheraP, and PSMAfore should not be interpreted interchangeably, because they addressed different clinical questions, enrolled different patient populations, applied different imaging-selection strategies, and used distinct control arms and endpoints (20–22). VISION established ^177Lu-PSMA-617 as a survival-prolonging therapy in a heavily pretreated post-ARPI and post-taxane mCRPC population, using standard of care as the control arm (20). However, the control arm excluded several active life-prolonging therapies, including chemotherapy, immunotherapy, ^223Ra, and investigational agents. This limits direct comparison with contemporary real-world treatment pathways, in which cabazitaxel, PARP inhibitors, or clinical trial options may be available (1, 3, 6, 20). Therefore, the OS benefit observed in VISION should be interpreted as evidence that ^177Lu-PSMA-617 improves outcomes over protocol-defined standard care in appropriately selected patients, rather than as proof of superiority over all active mCRPC therapies.

TheraP addressed a more direct comparative question by evaluating ^177Lu-PSMA-617 against cabazitaxel in patients considered suitable for taxane chemotherapy (21). This trial demonstrated higher PSA response rates and fewer grade 3–4 adverse events with ^177Lu-PSMA-617 than with cabazitaxel, suggesting that radioligand therapy may be preferable for selected patients with high PSMA expression and limited discordant disease (21). However, TheraP was a phase II trial powered for PSA response rather than OS, and its stringent dual-tracer imaging criteria selected a biologically favorable subgroup. In contrast to VISION, which required at least one PSMA-positive lesion and excluded predefined PSMA-negative lesions of clinically significant size, TheraP required high PSMA uptake and excluded patients with FDG-positive/PSMA-negative discordant lesions (20, 21). As a result, TheraP likely enriched the study population for tumors with high target expression and lower risk of early PSMA-independent progression. This difference in imaging selection is one of the most important reasons why response rates and outcomes from VISION and TheraP cannot be compared directly.

PSMAfore further changes the clinical interpretation of ^177Lu-PSMA-617 by evaluating it earlier in the mCRPC treatment sequence, before taxane exposure, in patients progressing after at least one ARPI (22). The significant rPFS improvement versus ARPI switching suggests that ^177Lu-PSMA-617 may become an earlier therapeutic option rather than being reserved exclusively for post-taxane disease (22). This is clinically important because sequential use of ARPIs after progression on a prior ARPI is generally associated with limited benefit due to cross-resistance, a phenomenon supported by molecular mechanisms such as androgen receptor pathway alterations and AR-V7 expression (41). In this context, PSMAfore supports the concept that PSMA-targeted radioligand therapy may provide a biologically distinct and clinically effective alternative to ARPI-to-ARPI switching. However, OS interpretation in PSMAfore is complicated by crossover, as many patients in the control arm received ^177Lu-PSMA-617 after progression (22). Consequently, the lack of a statistically significant OS advantage in the initial analysis should not be interpreted as absence of survival benefit; rather, it reflects the methodological challenge of evaluating OS when an effective therapy is available after progression in the control group.

These trial differences have direct implications for real-world practice. In patients with progressive mCRPC after ARPI and docetaxel, ^177Lu-PSMA-617 is supported by VISION as a life-prolonging option, particularly when PSMA PET/CT demonstrates adequate target expression and there is no clinically significant PSMA-negative disease (20). In patients eligible for cabazitaxel, TheraP suggests that ^177Lu-PSMA-617 may offer superior biochemical response and better tolerability, but this conclusion is most applicable to patients meeting strict imaging criteria, especially those without FDG-positive/PSMA-negative lesions (21). In taxane-naive patients progressing after ARPI, PSMAfore supports earlier use of ^177Lu-PSMA-617 as an alternative to ARPI switching, although local regulatory status, previous therapies, treatment availability, and patient preference remain important determinants of sequencing (22).

Alpha-emitting radiopharmaceuticals provide a mechanistically distinct approach due to their high LET and very short path length, generally below 100 μm, resulting in dense and often irreparable double-strand DNA breaks with limited collateral damage. ^223Ra dichloride, which selectively localizes to areas of increased bone turnover, demonstrated improved OS compared with placebo and delayed skeletal-related events in the ALSYMPCA trial in patients with symptomatic bone-predominant mCRPC (26). However, its co-administration with abiraterone and prednisone was associated with an increased fracture risk, underscoring the importance of appropriate patient selection, bone-protective strategies, and treatment sequencing (27). Unlike PSMA-targeted agents, ^223Ra is not a tumor cell-directed radioligand but a bone-seeking alpha-emitter, and its role remains largely limited to patients with symptomatic bone-dominant disease without visceral metastases.

^225Ac-PSMA represents a highly potent PSMA-directed alpha-emitting therapy. Early-phase clinical studies have reported substantial biochemical responses, suggesting that alpha-therapy may overcome selected resistance mechanisms associated with beta-emitting radioligands (32–36, 41). The biological rationale is strong: the high LET and short path length of alpha particles may be advantageous for small-volume disease, micrometastatic lesions, or tumors less responsive to beta-emitting therapy. Nevertheless, ^225Ac-PSMA remains an investigational modality and is not currently approved for routine clinical use in the management of mCRPC (40). The existing literature is largely derived from early-phase, retrospective, and heterogeneous studies with relatively small patient cohorts. To date, there are no completed randomized phase III trials comparing ^225Ac-PSMA against ^177Lu-PSMA-617 or standard taxane-based chemotherapy. Without such high-level evidence, it cannot be concluded that ^225Ac provides an OS benefit superior to established standard-of-care therapies. Completion of randomized phase III trials is required to validate both the safety and efficacy of ^225Ac-PSMA.

Several barriers currently limit broader implementation of ^225Ac-PSMA therapy. First, individualized dose optimization remains challenging. Treatment planning requires assessment of absorbed radiation doses in critical organs, particularly kidneys, salivary glands, and bone marrow (42). Accurate dosimetry for alpha emitters is intrinsically difficult because of the extremely short tissue range of alpha particles, the high spatial heterogeneity of energy deposition, and the limitations of standard imaging modalities used for treatment planning. Second, ^225Ac decay produces daughter isotopes that may redistribute *in vivo* and accumulate outside the intended therapeutic target, potentially causing off-target organ toxicity (31, 37). Third, toxicity remains a key limitation. Xerostomia, attributable to physiological PSMA expression in salivary glands, is more frequent and often more clinically limiting than with ^177Lu-based therapy, with a meaningful impact on patient-reported QoL (33, 37–42). Hematologic toxicity, although generally manageable, requires careful monitoring, particularly in heavily pretreated patients with compromised marrow reserve or extensive skeletal metastatic disease (33, 37–42). Finally, the global availability of medical-grade ^225Ac remains severely restricted. Production methods include ^229Th/^225Ac generator systems, irradiation of ^226Ra targets, and proton irradiation of ^232Th in cyclotrons. A photoneutron reaction of ^226Ra may generate ^225Ra, which subsequently decays to ^225Ac; however, these production routes remain technically demanding and are not yet widely accessible (29, 30, 37). These factors collectively explain why ^225Ac-PSMA remains largely confined to clinical trials, early-phase studies, and selected salvage settings.

Patient selection remains one of the most critical determinants of clinical outcomes in PSMA-targeted radioligand therapy. To optimize efficacy and minimize toxicity, clinicians must evaluate a complex matrix of molecular imaging, organ function, disease burden, and clinical history (6, 11, 20). Eligibility criteria based on PSMA PET/CT vary significantly between pivotal trials. In VISION, PSMA-positive status was determined using centrally read ^68Ga-PSMA-11 PET/CT alongside diagnostic-grade CT. Eligible patients were required to have at least one metastatic lesion demonstrating radiotracer uptake greater than that of normal liver parenchyma, regardless of lesion size. Crucially, the protocol excluded patients with specific PSMA-negative lesions, defined as uptake equal to or lower than liver background, that met predefined morphological thresholds. Patients were deemed ineligible if such PSMA-negative uptake was observed in any lymph node ≥2.5 cm in the short axis, any solid-organ metastasis ≥1.0 cm in the short axis, or any bone metastasis with a soft-tissue component ≥1.0 cm (20). In contrast, TheraP adopted more stringent dual-tracer imaging criteria. Patients were required to demonstrate high PSMA expression, defined as SUVmax ≥20 in at least one lesion and SUVmax >10 across all measurable sites. Furthermore, TheraP used ^18F-FDG PET/CT to exclude patients with spatially discordant disease, meaning FDG-positive but PSMA-negative lesions. This distinction is clinically important because FDG-avid/PSMA-negative metastases represent a dedifferentiated and aggressive phenotype associated with early treatment failure and unfavorable prognosis (21).

Eligibility for ^177Lu-PSMA-617 radioligand therapy extends beyond confirmation of PSMA-positive disease on PET/CT. In the pivotal VISION trial, candidates were required to have progressive mCRPC after prior treatment with at least one ARPI and one taxane-based chemotherapy regimen, adequate performance status, preserved bone marrow reserve, and sufficient renal and hepatic function. Typical laboratory requirements included adequate hemoglobin, neutrophil, and platelet counts, serum creatinine ≤1.5× the upper limit of normal or creatinine clearance ≥50 mL/min, and acceptable liver function parameters. Importantly, patients with PSMA-negative lesions of clinically significant size were excluded, highlighting the importance of sufficiently homogeneous PSMA expression for optimal treatment efficacy. These criteria reflect the need to balance treatment tolerability with the likelihood of therapeutic benefit (20).

However, anatomical distribution and total disease burden must also be carefully assessed. Metastatic spread to visceral organs, particularly the liver, is a well-documented negative prognostic factor associated with aggressive and dedifferentiated tumor biology. This was demonstrated in the large international multicenter study by Gafita et al., which analyzed 529 patients to develop and validate prognostic nomograms for OS and PSA-PFS following ^177Lu-PSMA-617 therapy. These nomograms underscore that patient selection cannot rely on imaging positivity alone. Rather, molecular imaging data should be systematically integrated with baseline clinical and laboratory parameters. The study demonstrated that survival after PSMA-targeted radioligand therapy is influenced not only by traditional indicators of disease aggressiveness but also by quantitative and anatomical features derived from PSMA PET/CT imaging (56).

In the OS model, several baseline factors were independently associated with poorer outcomes, including shorter interval between initial prostate cancer diagnosis and initiation of ^177Lu-PSMA-617 therapy, prior exposure to taxane chemotherapy, lower baseline hemoglobin concentration, extensive bone involvement, liver metastases, higher number of PSMA-positive metastatic lesions, and lower tumor PSMA expression reflected by SUV_mean_ on baseline PSMA PET/CT. The association between shorter disease duration and inferior OS likely reflects more aggressive tumor biology characterized by rapid progression to castration resistance. Similarly, prior chemotherapy should not be interpreted as a detrimental factor *per se*, but rather as a surrogate marker of advanced disease burden, treatment resistance, and cumulative biological aggressiveness. Baseline hemoglobin emerged as one of the strongest clinical prognostic variables for OS. Lower hemoglobin levels may reflect extensive bone marrow infiltration, chronic systemic inflammation, nutritional deterioration, or impaired hematopoietic reserve. Importantly, anemia may also reduce tolerance of subsequent systemic therapies and therefore influence survival independently of tumor progression itself. Notably, hemoglobin remained significant in the OS model despite adjustment for imaging-derived tumor burden, suggesting that it captures aspects of patient fitness and host reserve not reflected by imaging alone (56).

Among imaging variables, disease distribution had major prognostic implications. Bone involvement and particularly liver metastases were associated with significantly worse OS. Liver metastases are widely recognized as a marker of aggressive disease biology in mCRPC and have consistently been associated with poor survival across multiple therapeutic settings, including ARPI therapy, chemotherapy, and radioligand therapy. Their inclusion in the nomogram emphasizes that anatomical patterns of metastatic spread retain prognostic significance even in the era of molecular imaging-guided treatment. The total number of PSMA-positive metastatic lesions also independently predicted OS, highlighting the importance of global tumor burden rather than merely the presence of metastatic disease. Patients with extensive PSMA-avid disease exhibited substantially shorter survival, suggesting that increasing tumor volume may exceed the therapeutic capacity of the radiation dose delivered by ^177Lu-PSMA-617. Consequently, quantitative assessment of disease burden on PSMA PET provides clinically relevant prognostic information beyond conventional staging systems. An especially important observation was the prognostic role of tumor PSMA expression: higher SUV_mean_ values on baseline PSMA PET/CT were associated with improved OS. Although elevated PSMA expression has traditionally been linked to aggressive prostate cancer biology, in the context of PSMA-directed radioligand therapy it simultaneously provides a more favorable therapeutic target. Increased tracer uptake likely reflects greater ligand delivery and higher absorbed radiation doses within tumor lesions, thereby enhancing treatment efficacy. Thus, high PSMA expression may function as a predictive biomarker of treatment benefit despite its association with biologically aggressive disease (56).

For PSA-PFS, the final model shared several prognostic determinants with the OS model but also demonstrated notable differences. Factors associated with shorter PSA-PFS included shorter time since diagnosis, prior chemotherapy exposure, liver metastases, higher number of metastatic lesions, and lower tumor SUV_mean_. In contrast to the OS model, baseline hemoglobin was not retained as an independent predictor. This distinction is biologically plausible. PSA-PFS primarily reflects tumor responsiveness to radioligand therapy and the kinetics of biochemical progression, whereas OS is influenced by a broader spectrum of factors, including frailty, marrow reserve, comorbidities, and access to subsequent treatment lines. Consequently, hemoglobin may have a stronger effect on long-term survival than on immediate biochemical efficacy of ^177Lu-PSMA-617 therapy (56).

An additional noteworthy finding was the absence of ECOG performance status from the final prognostic models. Although ECOG is a well-established prognostic marker across advanced malignancies, it did not retain independent significance after adjustment for clinical and imaging variables. This may reflect both the relatively homogeneous performance status of patients selected for radioligand therapy and the superior prognostic value provided by quantitative PSMA PET-derived parameters. Collectively, these findings indicate that poor outcomes after ^177Lu-PSMA-617 therapy are driven by a combination of aggressive disease biology, high metastatic burden, unfavorable metastatic distribution, reduced hematologic reserve, and insufficient PSMA target expression. Conversely, patients with preserved hemoglobin levels, high tumor PSMA uptake, limited metastatic burden, and absence of visceral disease appear most likely to experience prolonged OS and PSA-PFS (56).

Patient selection for ^225Ac-PSMA targeted alpha therapy is less standardized than for ^177Lu-PSMA-617 and is not defined by prospective trial-based eligibility criteria. Instead, it derives from early-phase studies, retrospective series, and real-world clinical experience. Consequently, treatment decisions are highly individualized and are typically made in salvage settings for patients with mCRPC who have exhausted standard systemic treatment options, including ARPIs and, in many cases, prior ^177Lu-PSMA-617 radioligand therapy (39, 43, 46). Across published cohorts, eligibility generally includes adequate performance status, commonly ECOG 0–2, and sufficient baseline hematologic, renal, and hepatic function to permit safe administration of therapy. However, no universally accepted laboratory thresholds exist, and reported criteria vary between institutions and studies (39, 45, 46). Bone marrow reserve represents a key consideration, as many treated patients are heavily pretreated and frequently present with extensive skeletal metastatic disease. As a result, pre-existing cytopenias or impaired marrow function may increase the risk of cumulative hematologic toxicity (39, 43). Overall, ^225Ac-PSMA therapy is currently reserved for highly selected patients with advanced treatment-refractory mCRPC, in whom adequate organ function and marrow reserve are required to balance potential therapeutic benefit against the risk of significant toxicity, particularly xerostomia and hematologic adverse events (39, 43, 45).

Integrating dual-tracer molecular imaging with patient-specific clinical characteristics and comorbidities, together with emerging circulating biomarkers, may further refine patient selection for both beta- and alpha-emitting radioligand therapies. In particular, biomarkers such as circulating tumor DNA and AR-V7 status represent promising tools for capturing tumor heterogeneity and systemic treatment resistance beyond conventional imaging and laboratory parameters. Although these approaches are not yet incorporated into routine clinical decision-making, their combined use with PSMA-based molecular imaging may ultimately enable more comprehensive biological stratification of patients and improve the precision of radioligand therapy selection (41).

Comparatively, ^177Lu- and ^225Ac-based PSMA therapies occupy complementary rather than interchangeable positions within the therapeutic landscape. ^177Lu-PSMA-617 is supported by robust randomized evidence and currently represents the standard PSMA-directed radioligand therapy in mCRPC (6, 20–22). It may be particularly suitable for patients with heterogeneous but PSMA-positive disease because beta-particle crossfire can irradiate adjacent tumor cells with variable PSMA expression. In contrast, ^225Ac-PSMA has a shorter path length and higher LET, which may be advantageous for small-volume disease, micrometastatic lesions, or tumors resistant to beta-emitting therapy (31–39). However, the absence of randomized phase III evidence, limited radionuclide availability, dosimetric uncertainty, and higher burden of xerostomia currently restrict ^225Ac-PSMA to investigational or salvage contexts (34, 43, 46–48). Therefore, individualized treatment planning should consider PSMA expression intensity on PET/CT, disease burden and distribution, prior therapies, bone marrow reserve, renal and hepatic function, comorbidities, expected toxicity, and patient preference.

Treatment sequencing after prior ARPI, taxane, PARP inhibitor, immunotherapy, or radioligand exposure remains unresolved and should be individualized. After ARPI progression, switching to another ARPI is generally less attractive than introducing a mechanistically distinct therapy, such as taxane chemotherapy, PARP inhibition in patients with actionable homologous recombination repair alterations, or PSMA-targeted radioligand therapy in PSMA-positive disease. After docetaxel, the choice between cabazitaxel and ^177Lu-PSMA-617 should consider PSMA expression intensity, FDG/PSMA discordance when available, marrow reserve, visceral disease, symptoms, prior toxicities, and patient preference (21, 56). PARP inhibitors should be prioritized in molecularly selected patients with relevant DNA repair defects, whereas immunotherapy remains restricted to small biomarker-defined subgroups, such as MSI-high or mismatch repair-deficient disease (1, 3, 6). Following prior ^177Lu-PSMA-617 exposure, rechallenge may be considered in selected patients with prior durable benefit and persistent PSMA expression, while ^225Ac-PSMA or tandem alpha-beta strategies remain investigational options that may be considered mainly in clinical trials or highly selected salvage settings (43, 55).

Beyond monotherapies, the combination of ^225Ac-PSMA and ^177Lu-PSMA-617, frequently referred to as tandem alpha-beta radioligand therapy, represents a promising strategy for selected patients with advanced mCRPC. This approach may be particularly relevant for patients who have progressed on or exhausted conventional ^177Lu-PSMA-617 monotherapy. Mechanistically, tandem therapy aims to exploit the high LET of alpha particles to overcome radiation resistance while potentially mitigating the severe salivary gland toxicity associated with full-dose ^225Ac regimens. By de-escalating ^225Ac activity and complementing it with the longer-range beta emissions of ^177Lu, salivary gland exposure may be partially reduced while preserving antitumor activity. The clinical efficacy of this approach was recently quantified in a systematic review and meta-analysis by Belabaci et al., which demonstrated a pooled PSA decline of ≥50% in 47% of patients and a median OS of 11.8 months in heavily pretreated cohorts (57). However, the evidence remains heterogeneous and non-randomized, and tandem therapy should therefore be considered investigational rather than a standardized treatment strategy.

The widespread clinical implementation of tandem therapy and alpha-emitting PSMA approaches also faces logistical bottlenecks, most notably the constrained global supply of ^225Ac, as reviewed by Yamamichi et al. (58). Looking forward, the therapeutic horizon is expanding toward combining radioligands with complementary systemic agents, including PARP inhibitors, taxanes, ARPIs, and immune checkpoint inhibitors. Preclinical evidence suggests that high-LET alpha radiation may remodel the tumor microenvironment, increase tumor immunogenicity, and potentially enhance the efficacy of immunotherapy (58). Nevertheless, successfully refining these protocols will require standardized imaging selection, prospective comparative trials, optimized dosimetry, toxicity mitigation, and robust predictive biomarkers to determine which patients benefit most from beta-emitting, alpha-emitting, tandem, or combination-based radioligand strategies. Therefore, future research should focus on defining evidence-based sequencing algorithms, validating biomarkers of response and resistance, improving management of salivary and hematologic toxicity, and determining the optimal integration of radioligand therapy within the broader therapeutic landscape of mCRPC (29, 30, 37–42).

## 6 Conclusion

PSMA-targeted radioligand therapy represents one of the most important therapeutic advances in the management of mCRPC over the past decade. Nevertheless, both primary and acquired resistance remain clinically significant challenges. Tumor heterogeneity in PSMA expression, clonal evolution driven by therapeutic pressure, and the emergence of neuroendocrine differentiation may contribute to treatment failure. These mechanisms highlight the need for predictive biomarkers and improved strategies for treatment sequencing. The future role of PSMA-directed radioligand therapy will depend not only on the development of more effective radionuclides but also on its integration into treatment algorithms. Precise selection of patients, adequate sequencing of therapies, and the development of rational combination approaches will be critical in determining whether radioligand therapy evolves from a later-line option to a central component of prostate cancer management. As the field of theranostics continues to mature, PSMA-targeted radioligand therapy represents a compelling example of precision oncology, combining molecular imaging (PSMA PET/CT) with targeted cytotoxic treatment within a single therapeutic framework. Ongoing clinical and translational studies will further clarify its optimal positioning within treatment paradigms and its long-term impact on survival outcomes and quality of life in patients with mCRPC.
